# Pre-Stimulus Sham TMS Facilitates Target Detection

**DOI:** 10.1371/journal.pone.0057765

**Published:** 2013-03-04

**Authors:** Felix Duecker, Alexander T. Sack

**Affiliations:** 1 Department of Cognitive Neuroscience, Faculty of Psychology and Neuroscience, Maastricht University, Maastricht, The Netherlands; 2 Maastricht Brain Imaging Center, Faculty of Psychology and Neuroscience, Maastricht University, Maastricht, The Netherlands; Radboud University Nijmegen, The Netherlands

## Abstract

Transcranial magnetic stimulation (TMS) allows non-invasive manipulation of brain activity during active task performance. Because every TMS pulse is accompanied by non-neural effects such as a clicking sound and somato-sensation on the head, control conditions are required to ensure that changes in task behavior are indeed due to the induced neural effects. However, the non-neural effects of TMS in the context of a given task performance are largely unknown and, consequently, it is unclear what constitutes a valid control condition. We explored the non-neural effects of TMS on visual target detection. Participants received single pulse sham TMS to each hemisphere at different time points prior to target appearance during a visual target detection task. It was hypothesized that the clicking sound of a sham TMS pulse differentially affects performance depending on the location of the coil and the timing of the pulse.Our results show that, first, sham TMS caused a facilitation of reaction times when preceding the target stimulus by 150, 200, and 250 ms, whereas earlier and later time windows were not effective. Second, positioning the TMS coil ipsilateral instead of contralateral relative to the target stimulus improved reaction times. Third, infrequent noTMS trials that were interleaved with sham TMS trials had oddball-like properties resulting in increased reaction times during noTMS. The clicking sound produced by sham TMS influences task performance in multiple ways. These non-neural effects of TMS need to be controlled for in TMS research and the present findings provide an empirical basis for deciding what constitutes a valid control condition.

## Introduction

Transcranial magnetic stimulation (TMS) is a widely used method in neuroscience that allows non-invasive manipulation of brain activity in healthy human volunteers by exposing the brain to a rapidly changing magnetic field. Next to the intended neural effects of TMS, every TMS pulse produces a distinct clicking sound and sensations on the head, and thus “non-neural” side effects, that potentially also systematically influence task behavior. Obviously, there is a strong need for appropriate control conditions in order to ensure that effects of interest are indeed the result of direct TMS-induced brain activity changes and not a consequence of the non-neural side effects of TMS. Before going into further detail, we would like to comment on the terminology introduced here. The dichotomy of ‘neural’ versus ‘non-neural’ effects could be regarded as being misleading or even incorrect. Of course, the clicking sound of the TMS coil is processed by the brain. In this sense, all behavioral effects caused by a TMS pulse are ultimately neural, no matter how they arise. However, when focusing on the immediate (physical) properties of a TMS pulse, ‘neural’ points to the changes in brain activity as they are caused by the magnetic field of the TMS pulse. In contrast, ‘non-neural’ is meant to refer to the sound and vibration of the TMS coil which are not per se related to neural events in the brain.

There are different strategies of controlling for non-neural effects of TMS and the choice of control conditions often depends on the research question and experimental design [1]. In general, the idea is to demonstrate the specificity of results so that the same outcome is not observed under different TMS conditions. To give a few examples, TMS is commonly applied to multiple brain areas in order to show that the results are specific to one stimulation site. This can either be achieved by stimulation of brain areas that are differentially involved in a particular task or process, or by comparing the experimental condition to TMS over the vertex which should have no neural effect (spatial specificity). Similarly, when applying TMS at different time points, temporal information about the involvement of brain areas can be obtained aiming to reveal that the observed effects depend on the precise timing of stimulation (temporal specificity). Finally, there are sham TMS coils that mimic real TMS coils by producing the same clicking sound, and sometimes even sensations on the head, but do not cause any change in neural activity. Again, the idea is that a comparison between sham TMS and real TMS reveals the unique contribution of the neural effect of TMS on behavior. Importantly, the underlying assumption in all these cases is that the non-neural side effects of TMS are unspecific and therefore do not cause the behavioral differences between conditions. Although this assumption is vital for the interpretation of many TMS findings, only few studies have investigated the non-neural effects of TMS on behavior.

Regarding temporal specificity, there are few studies that report intersensory facilitation when a TMS pulse is administered in close temporal proximity to a target stimulus [2], [3], [4]. This indicates that the clicking sound of a TMS pulse can indeed confound results when comparing different time points of stimulation. Similarly, subjective ratings of discomfort due to TMS have been found to positively correlate with error rate on a working memory task [5]. However, to the best of our knowledge, other potential side effects have not been investigated yet. For the most part, it therefore appears an open empirical question whether the non-neural TMS side effects such as clicking sound of the TMS coil are indeed unspecific for task performance. This makes it difficult to decide on an empirical basis what constitutes a valid control condition. Next to the effects mentioned above, it might very well be that also spatial and other temporal non-neural aspects of a TMS pulse selectively modulate task performance and thus create behavioral specificity that could then be falsely interpreted as a direct neural effect of TMS, i.e. a false positive finding. Similarly, such a potential behavioral specificity of the non-neural effects of TMS might also work against the intended TMS-induced neural effects, leading to false null findings.

Here, we explored the non-neural effects of TMS on visual target detection. To this end, we applied sham TMS over the left and right hemisphere at different time points prior to target appearance. We aimed to address three basic empirical questions that have direct relevance for many TMS studies and provide guidance in designing appropriately controlled TMS experiments. First, we investigated whether a sham TMS pulse serves as a warning signal that transiently facilitates target detection. Many studies have shown that auditory stimuli can improve subsequent detection of visual targets in particular due to two different processes, namely temporal orienting and changes in phasic arousal [6], [7]. We hypothesized that the clicking sound of an appropriately timed TMS pulse has a similar effect as reflected by decreased reaction times for visual target detection. Second, we explored the possibility that a lateralized TMS pulse automatically pulls spatial attention to the corresponding side of space. Almost all TMS studies apply TMS to brain areas away from the mid-sagittal line and, consequently, the clicking sound can be perceived as originating from one hemifield. Analogous to a cross-modal exogenous cueing task, we hypothesized that a TMS pulse facilitates target detection in the ipsilateral hemifield due to stimulus-driven shifts of spatial attention [8], [9], [10]. Finally, it is common to include trials without TMS that are sometimes used as a control condition even though they might not reflect proper baseline performance. We hypothesized that performance on noTMS trials is context-dependent. Infrequent noTMS trials that are interleaved with sham TMS trials are unexpected and might have oddball-like properties resulting in increased reaction times compared to blocked noTMS trials [11], [12]. Taken together, sham TMS is hypothesized to specifically influences task behavior in multiple ways despite the absence of neural effects and, consequently, can reveal factors that need to be controlled for when conducting real TMS experiments in order to avoid false positive or false negative findings.

## Methods and Materials

### Ethics Statement

The study was approved by the medical-ethical committee of the University Medical Center, Maastricht, the Netherlands. All participants gave written informed consent prior to participation and were screened for TMS experimentation safety by a medical supervisor.

### Participants

Eighteen participants (12 female, aged 19 to 26) were recruited from the student population of Maastricht University. All had normal or corrected-to-normal vision and were right-handed. The research question and hypotheses remained unknown to the participants until the end of the experiment.

### Stimuli and Task

**Figure 1:**
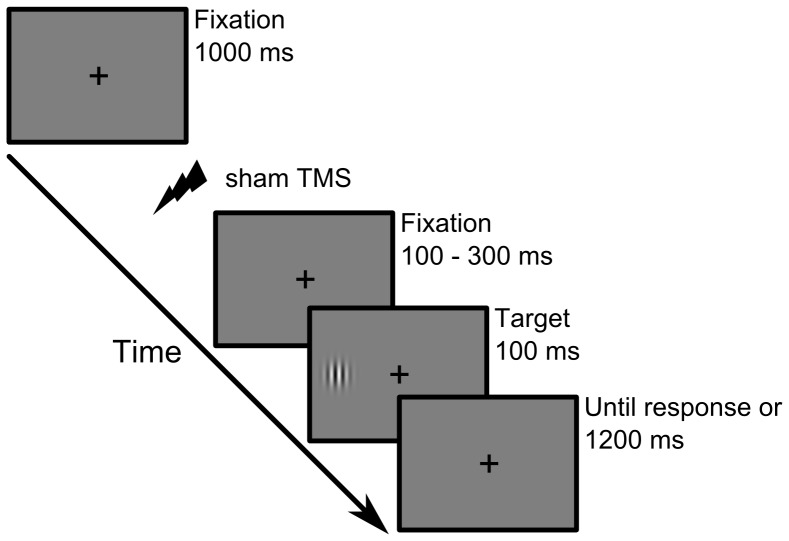
Example of a single trial of the detection task. Sham TMS was applied prior to target appearance and the time interval between TMS pulse and target onset was variable across trials ranging from 100 to 300 ms. Target stimuli were briefly flashed either in the left or right hemifield and participants were instructed to respond as fast as possible with a single button press.

Participants performed a simple detection task requiring a single button press whenever a target stimulus was presented irrespective of target location (see Figure 1). A fixation cross was continuously presented at the centre of the screen and Gabor patches served as target stimuli that were shown for 100 ms either left or right of the fixation cross at 7 degrees eccentricity (spatial frequency = 1.5 cycles per degree; envelope standard deviation = 0.75 degrees; Michelson contrast = 60%; random orientations). Participants were instructed to press the space bar on a standard keyboard with the right index finger as fast as possible as soon as the target stimulus appeared. Corrective feedback (an error message on the screen) was given in case of false alarms, misses, anticipatory responses (RT below 100 ms), or very slow responses (RT above 800 ms). Stimuli were presented on a gamma-corrected 17′′ TFT screen (Samsung SyncMaster 931 DF) at 57 cm viewing distance with the head supported by a chin rest. The video mode was 1280×1024 at 60 Hz and background luminance was 25 cd/m^2^. The Presentation software package (NeuroBehavioural Systems, Albany, CA) was used to control stimulus presentation and recording of behavioral responses.

### Procedure and Design

The experiment consisted of one session per participant based on a full within-subject design. During sham TMS trials, a single pulse was delivered to the right or left hemisphere at 300, 250, 200, 150, or 100 ms prior to target appearance. The exact position of the TMS coil on each participant’s head was defined based on the International 10–20 system, namely C3 and C4. The TMS pulse contained no information about where the target stimulus would appear and catch trials were included to reduce anticipatory responses. We also included trials without TMS that were interleaved with sham TMS trials. For both stimulation sites, participants completed five blocks each consisting of 56 trials in randomized order resulting in 20 trials for each condition (including noTMS trials) and 40 catch trials. The TMS coil position was switched after five blocks and the order of conditions was counterbalanced across participants. Additionally, a single block of 80 trials without TMS was presented halfway through the experiment. After each block, participants could take a short break and received feedback about their average reaction time to ensure that they were motivated throughout the session.

### Transcranial Magnetic Stimulation

Sham TMS pulses were applied using a Medtronic MagPro X100 stimulator (Medtronic Functional Diagnostics A/S, Skovlunde, Denmark) and a figure-of-eight placebo TMS coil (MC-P-B70; inner radius = 10 mm; outer radius = 50 mm). This coil is identical to a real TMS coil so that it produces the same clicking sound when discharging but a magnetic shield reduces the effective magnetic field by approximately 80%. Stimulation intensity was set at 30% maximum stimulator output for all participants. At this intensity, a sham TMS pulse is too weak to produce any neural effect and hardly any sensation on the head can be perceived, except for weak vibrations of the coil. Participant received sham TMS over the left and right hemisphere, that is electrode position C3 and C4, respectively. Using a mechanical arm, the TMS coil was placed perpendicular on the head with the handle pointing posterior and slightly lateral.

### Eye Movement Control

In order to exclude trials with breaks of central fixation, eye movements and eye blinks were measured using electrooculography (EOG). Data were recorded bipolarly from two pairs of Ag/AgCl electrodes with a BrainAmp ExG system (BrainProducts GmbH, Munich, Germany). Electrodes were positioned at the outer canthus of each eye (horizontal EOG), above and below the right eye (vertical EOG), and on the mastoid behind the right ear (reference). The impedance of all electrodes was kept below 5 kΩ. The EOG signal was digitized at 1000 Hz, high-pass filtered at 0.1 Hz, and stored on disk using BrainVision Recorder (BrainProducts GmbH, Munich, Germany). Offline data analysis was performed with BrainVision Analyzer (BrainProducts GmbH, Munich, Germany). Single trial data were visually inspected and all trials contaminated by eye movements or eye blinks were excluded from further analysis.

### Statistical Analysis

After exclusion of all incorrect trials, outliers were removed from the data using the 1.5×IQR (interquartile range) criterion. Individual mean reaction times were then submitted to repeated measures ANOVAs. Depending on the hypothesis being tested, within-subject factors were sham TMS time window (5 levels: −300, −250, −200, −150, −100), TMS coil position (2 levels: ipsilateral, contralateral), target location (2 levels: left, right), or context of no TMS trials (2 levels: interleaved, blocked). Effects of interest were then further explored with post-hoc contrasts and paired t-tests with correction for multiple comparisons by Fisher’s LSD procedure.

## Results

### Errors and Eye Movements

All participants were able to perform the task at high levels of accuracy. Targets were correctly detected in 98.8% of all trials and false alarms only occurred in 4.4% of the catch trials. A one-way repeated-measures ANOVA with sham TMS time window as within subject factor revealed a significant effect on accuracy (*F*_(4,14)_ = 6.395, *p* = .004). Post-hoc analysis showed a significant linear contrast (*F*_(1,17)_ = 24.232, *p*<.001) resulting from decreasing accuracy with longer durations between sham TMS and target appearance due to slightly higher numbers of premature responses. Given that participants sometimes fail to withhold their response in anticipations of the target stimulus, this is not surprising because the likelihood of doing so increases with the length of the time interval. Nevertheless, accuracy was very high in all TMS time windows and never dropped below 96.9% on the group level. Similarly, accuracy in the interleaved and blocked trials without TMS was 99.4% and 99.0%, respectively, and a paired samples t-test showed no significant difference (*t*_(17)_ = .652, *p* = .523). In order to exclude any confounding effect of eye movements or eye blinks on reaction times, all trials with breaks of central fixation were discarded. In total, only 4.0% of all trials were contaminated ranging from 0.2% to 15.4% across participants. In sum, this indicates that participants had no difficulties performing the task and did not trade speed for accuracy so that the remaining analyses focused on reaction time differences.

### No TMS Trials

Target detection in the absence of TMS was performed in two different contexts, either interleaved with sham TMS or as a separate noTMS block. A repeated-measures ANOVA on mean reaction times with context (interleaved, blocked) and target location (left, right) as within-subject factors revealed a highly significant main effect of context (*F*_(1,17)_ = 248.257, *p*<.001), no effect of target location (*F*_(1,17)_ = 0.197, *p* = .663), and no interaction (*F*_(1,17)_ = 0.007, *p* = .933). The main effect of context resulted from slower reaction times for noTMS trials that were interleaved with sham TMS (*M* = 377 ms, *SE* = 8.9) as compared with blocked noTMS trials (*M* = 295 ms, *SE* = 8.0). This shows that noTMS trials do not necessarily reflect undistorted baseline performance but are modulated by context. In our case, when noTMS trials are interleaved with sham TMS they become infrequent events that have oddball–like properties (only one out of six targets was not preceded by a sham TMS pulse) resulting in slower reaction times. For that reason, only the blocked noTMS trials were used in subsequent analyses as a comparison for sham TMS related changes in target detection.

### Sham TMS Trials

We then examined the effects of sham TMS on reaction times. A repeated-measures ANOVA with sham TMS time window (−300, −250, −200, −150, −100), TMS coil position (ipsilateral, contralateral), and target location (left, right) as within-subject factors revealed significant main effects of sham TMS time window (*F*_(4,14)_ = 9.548, *p*<.001), TMS coil position (*F*_(1,17)_ = 25.615, *p*<.001), and target location (*F*_(1,17)_ = 5.528, *p* = .031). There were no significant interactions (all *p* values >.25).

**Figure 2:**
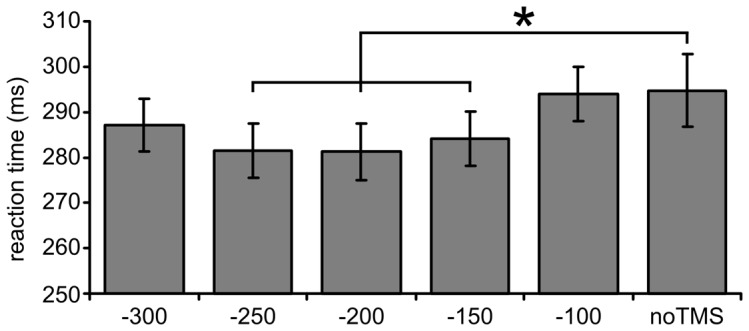
The effect of sham TMS time window on mean reaction time. A transient facilitation of reaction times was observed for intermediate time windows compared to blocked noTMS trials. Error bars show the standard error of the mean.

The main effect of sham TMS time window was best accounted for by a highly significant quadratic contrast (*F*_(1,17)_ = 30.177, *p*<.001), resulting from an U-shaped reaction time curve, that is, intermediate time windows showed decreased reaction times relative to the earliest and latest time window. The same pattern of results was obtained when comparing all TMS time windows with blocked noTMS trials (Figure 2). Post-hoc pairwise comparisons revealed significantly decreased reaction times when the sham TMS pulse preceded the target by 250 ms (*t*_(17)_ = 2.673, *p* = .016), 200 ms (*t*_(17)_ = 2.667, *p* = .016), and 150 ms (*t*_(17)_ = 2.507, *p* = .023). There was no significant effect of the earliest (*t*_(17)_ = 1.611, *p* = .126) and latest time window (*t*_(17)_ = 0.131, *p* = .897), that is, 300 ms and 100 ms prior to target appearance, respectively. This suggests that a sham TMS pulse serves as a warning stimulus that transiently facilitates target detection.

**Figure 3:**
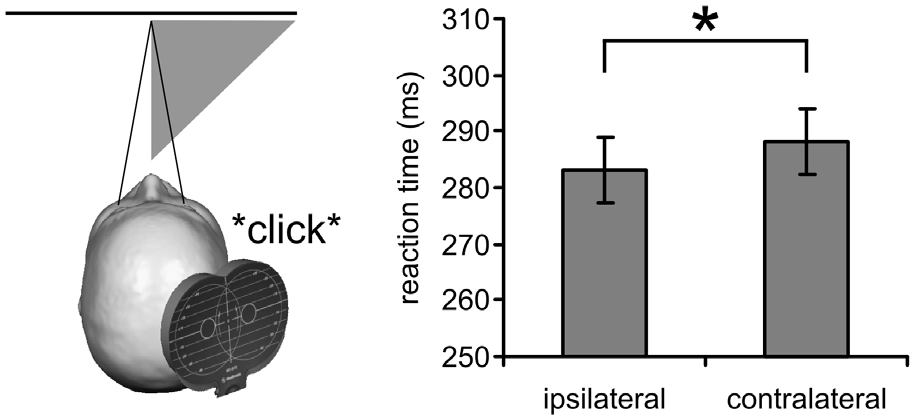
The effect of TMS coil position on mean reaction time. The clicking sound of a TMS pulse caused a shift of covert spatial attention to the corresponding side of space thereby facilitating target detection. Error bars show the standard error of the mean.

The main effect of TMS coil position resulted from faster reaction times when the sham TMS coil was ipsilateral (*M* = 283 ms, *SE* = 5.8) instead of contralateral (*M* = 288 ms, *SE* = 5.9) to the target stimulus (Figure 3). This indicates that a lateralized sham TMS pulse causes a reflexive shift of covert spatial attention to the corresponding side of space and thereby facilitating target detection.

Finally, the main effect of target location was caused by faster reaction times for target stimuli in the right hemifield (*M* = 284 ms, *SE* = 6.1) compared with the left hemifield (*M* = 288 ms, *SE* = 5.8) probably because participant used their right hand to respond (Simon effect, [13]).

## Discussion

The non-neural effects of TMS on behavior are largely unknown and, consequently, it is unclear what constitutes a valid control condition. It is often assumed that the used control conditions show no behavioral specificity in the context of a given experimental task. Surprisingly, this assumed lack of specificity of, e.g., sham TMS has rarely been tested empirically. Here, we investigated the effects of pre-stimulus sham TMS on target detection and provide evidence that sham TMS produces systematic and specific changes in target detection reaction times. Depending on the experimental design and research question, these specific effects of sham TMS can lead to false positive or false negative results. Our findings thus have direct implications for many TMS experiments, and could be used to guide the choice of an appropriate control condition in the context of a concrete research question. Specifically, we tested three hypotheses of sham TMS-induced behavioral specificity that were all confirmed by our present findings.

Our first hypothesis was that an appropriately timed sham TMS pulse serves as an acoustic warning stimulus that facilitates visual target detection due to temporal orienting and/or increased levels of phasic arousal. Indeed, our results show decreased reaction times when a sham TMS pulse precedes the target stimulus by 150, 200, or 250 ms compared to our baseline condition whereas earlier and later time windows were not effective. The absence of an effect at 100 and 300 ms prior to target appearance suggests that the warning signal effect was mainly due to temporal orienting because of the following reasons. Once the sham TMS pulse is administered, the probability of target occurrence increases over time and, consequently, reaction times gradually become faster with increasing stimulus onset asynchronies (SOA). At 100 ms, the target stimulus might still come as a surprise and the time to prepare for target appearance might be insufficient, explaining the absence of an effect. Following this line of thought, reaction times at the 300 ms time window should be fastest but the inclusion of catch trials in the current experiment increased uncertainty for the longest SOAs because the target might not be presented at all resulting in slower reaction times. Taken together, the present pattern of results seems to be best accounted for in terms of temporal orienting. However, a transient effect on phasic arousal cannot be excluded and might contribute to the observed facilitatory effects. This strongly suggests that the clicking sound of a TMS pulse acts as a warning signal and has similar properties to other kinds of warning stimuli [6], [7]. This finding complements earlier reports of intersensory facilitation that occurs when the clicking sound of the TMS pulse temporally coincides with target appearance [2], [3], [4]. The time windows used in the present experiment were all prior to target appearance so that a direct comparison with these experiments is difficult. However, the absence of any facilitation at 100 ms before target onset suggests that the effects observed in our study do not reflect the same underlying mechanism. As argued above, an interpretation in terms of temporal orienting seems most likely. As a consequence, TMS experiments that compare different time points of stimulation cannot assume that the non-specific effects of TMS on behavior are constant across temporal conditions. Time-dependent TMS effects therefore require a control condition that consists of either real or sham TMS at the same time points as the experimental condition. Only then the non-neural effects of TMS on temporal orienting and/or arousal are actually controlled for.

Our second hypothesis was that a sham TMS pulse can cause an automatic shift of covert spatial attention when applied away from the mid-sagittal line, thereby facilitating unilateral target detection. Indeed, improved reaction times were observed when the TMS coil was positioned ipsilateral instead of contralateral relative to the target stimulus. This is in agreement with findings from cross-modal cueing tasks where non-predictive lateralized auditory stimuli have been shown to speed detection of visual targets at the cued location [8], [9], [10]. In many cases, a distance effect is observed with cueing effects being stronger when the precise location of the auditory cue and visual target match. Our results show that cross-modal cueing effects even occur in case of spatial correspondence regarding hemifields. As a consequence, TMS experiments that compare different stimulation sites cannot assume that the non-neural effects of TMS on behavior are constant across conditions. The cueing effect reported here can only be controlled for when the TMS coil is positioned at a location that produces the same effects on spatial attention. The conditions under which this is the case cannot be inferred from the present study because we only investigated the difference between the left and right hemisphere. Nevertheless, it seems necessary to choose a stimulation site as control condition that lies in the same hemisphere and maybe even has as a similar degree of laterality. This also implies that vertex stimulation is not a suitable control condition for lateral real stimulation sites especially when tasks require a rigid control of the locus of spatial attention.

Our third hypothesis was that performance on trials without TMS depends on the context in which they are presented. More specifically, infrequent noTMS trials that are interleaved with sham TMS trials were expected to have oddball-like properties [11], [12]. This was thought to result in increased reaction times compared to sham TMS trials and noTMS trials that are presented in separate blocks. Our results clearly show that reaction times on interleaved noTMS trials are strongly confounded and do not reflect proper baseline performance. Blocked noTMS trials, on the other hand, were indistinguishable from the earliest and latest sham TMS time window and, for our purposes, could be used for further analysis. In general, this strongly suggests that interleaved noTMS trials should not be used as baseline condition. They obviously do not control for the non-neural effects of TMS and, on top of that, are modulated by the presence of TMS trials. Since this modulation most likely depends on many aspects of the experimental design, the validity of noTMS trials as baseline condition is difficult to establish.

Taken together, our results provide insights into the specificity of non-neural effects of TMS on behavior. Importantly, this does not challenge the validity of sham TMS as a control condition. Quite the contrary, all the effects reported here most likely occur during real TMS as well. In this sense, our results demonstrate that sham TMS can be considered an appropriate control condition for at least some of the non-neural effects of TMS. However, the sham TMS coil used in this experiment does not produce sensations on the head that match those of real TMS. There might therefore be additional non-neural effects of TMS that remained undetected and require further investigation.

It has to be emphasized that the present study applied sham TMS always prior to target appearance. This makes our experiment particularly sensitive to the effects described above and it therefore remains unclear in how far similar non-neural effects of TMS can be observed with other stimulation parameters, tasks, and outcome measures. Of course, there is an abundance of TMS experiments that used sham TMS as a control condition and these could, in principle, be re-evaluated to discover additional non-neural side effects of TMS. While a full review of this matter is beyond the scope of this article, we examined previous publications from our own group in the light of the present findings. To begin with, reaction times on an angle judgment task were found to linearly increase across post-stimulus time windows when applying sham TMS suggesting that participant sometimes delay their response until a TMS pulse is administered [14]. Additionally, no TMS trials have repeatedly been associated with unusual slowing of reaction times [15], [16] resembling the context effect reported in the present study. However, there are also many studies that did not reveal changes in task behavior due to sham TMS. For example, pre-stimulus sham TMS over early visual cortex neither affected behavioral priming [17] nor did we observe any significant effects on accuracy and awareness ratings in a visual masking paradigm [18] whereas real TMS clearly revealed time-dependent modulations of task behavior. Similarly, Schuhmann et al. found no effect on picture naming latency when applying sham TMS over Broca’s area after stimulus presentation [19]. Overall, it seems that the non-neural side effects of TMS depend on many factors and the present study should be seen as a starting point for identifying those conditions that are especially vulnerable to such effects.

In conclusion, we show that the clicking sound produced by a sham TMS coil acts as a warning signal, causes automatic shifts of spatial attention, and creates a context that influences noTMS trials. These factors are relevant for a broad range of experiments and the present findings therefore provide an empirical basis for deciding what constitutes an appropriate control condition to avoid or minimize both, chances for false positive as well as false negative TMS findings.
